# Chronic Ouabain Prevents Radiation-Induced Reduction in the α2 Na,K-ATPase Function in the Rat Diaphragm Muscle

**DOI:** 10.3390/ijms231810921

**Published:** 2022-09-18

**Authors:** Violetta V. Kravtsova, Arina A. Fedorova, Maria V. Tishkova, Alexandra A. Livanova, Oleg V. Vetrovoy, Alexander G. Markov, Vladimir V. Matchkov, Igor I. Krivoi

**Affiliations:** 1 Department of General Physiology, St. Petersburg State University, 199034 St. Petersburg, Russia; 2 Pavlov Institute of Physiology, Russian Academy of Sciences, 199034 St. Petersburg, Russia; 3 Department of Biochemistry, St. Petersburg State University, 199034 St. Petersburg, Russia; 4 Department of Biomedicine, Aarhus University, C 8000 Aarhus, Denmark

**Keywords:** ionizing radiation, skeletal muscle, Na,K-ATPase isozymes, ouabain, resting membrane potential

## Abstract

The damaging effect of ionizing radiation (IR) on skeletal muscle Na,K-ATPase is an open field of research. Considering a therapeutic potential of ouabain, a specific ligand of the Na,K-ATPase, we tested its ability to protect against the IR-induced disturbances of Na,K-ATPase function in rat diaphragm muscle that co-expresses the α1 and α2 isozymes of this protein. Male Wistar rats (*n* = 26) were subjected to 6-day injections of vehicle (0.9% NaCl) or ouabain (1 µg/kg/day). On the fourth day of injections, rats were exposed to one-time total-body X-ray irradiation (10 Gy), or a sham irradiation. The isolated muscles were studied 72 h post-irradiation. IR decreased the electrogenic contribution of the α2 Na,K-ATPase without affecting its protein content, thereby causing sarcolemma depolarization. IR increased serum concentrations of ouabain, IL-6, and corticosterone, decreased lipid peroxidation, and changed cellular redox status. Chronic ouabain administration prevented IR-induced depolarization and loss of the α2 Na,K-ATPase electrogenic contribution without changing its protein content. This was accompanied with an elevation of ouabain concentration in circulation and with the lack of IR-induced suppression of lipid peroxidation. Given the crucial role of Na,K-ATPase in skeletal muscle performance, these findings may have therapeutic implications as countermeasures for IR-induced muscle pathology.

## 1. Introduction

Ionizing radiation (IR) has sufficient energy to ionize atoms by detaching electrons from them [1]. People are exposed to natural sources of IR from the surrounding environment on Earth and during space flights [2]; it is also widely used in research, industry, and medicine [3,4,5,6]. IR can present a health hazard when proper measures against excessive exposure are not taken [1]. Severe body irradiation as a result of accidents at nuclear power plants can lead to skin burns or acute radiation syndrome, whereas low doses of IR increase the risk of long-term effects, i.e., cancer [1]. IR causes cell damage to various organ systems and tissues, including skeletal muscles [3,6,7,8]. IR promotes the generation of reactive oxygen species (ROS), which further contributes to DNA damage [4], cell injury, and death. Moreover, an increased IL-6 signaling suggesting inflammation is also involved in IR pathology [9,10].

IR acts not only on DNA, but also on the plasma membrane, causing structural and functional damage to cell membranes and membrane-associated proteins, including ion channels and transporters, which can contribute to the death and survival of irradiated cells [11]. The negative effects of IR on Na,K-ATPase in various tissues have previously been reported [5,12,13]. IR-induced alterations may include both a decrease in enzyme activity [12,13] and a decrease in the membrane abundance of Na,K-ATPase [5]. The Na,K-ATPase pumps Na^+^ and K^+^ ions across the plasma membrane, which has a vital role in all living cells. This ion transport underlies the resting membrane potential (RMP), which is critical to the normal function of skeletal muscles [14], because this is important for Na^+^ channel function, membrane excitability, and excitation–contracting coupling [15,16,17]. Even a minor membrane depolarization but prolonged in time leads to inactivation of the Na^+^ channels, and thus, the suppression of membrane excitability that disrupts excitation–contracting coupling [15,16,17]. Skeletal muscles contain the largest pool of Na,K-ATPase in the body; therefore, IR-induced changes in the Na,K-ATPase may lead to dysfunction in muscle contraction [3,6,7]. However, this association remains to be elucidated.

In the skeletal muscle plasma membrane, α1 and α2 isoforms of the Na,K-ATPase are co-expressed [18,19]. In adult skeletal muscle, the α2 Na,K-ATPase isozyme comprises up to 87% of total content of the Na,K-ATPase protein [18,20]. In contrast to the α1 Na,K-ATPase, the α2 Na,K-ATPase isozyme has been proposed to be adaptive and dynamically regulated by muscle use, which enables working muscles to maintain excitability and resistance to fatigue [21,22]. The α1 Na,K-ATPase is relatively uniformly distributed in the sarcolemma and serves householding functions [23]. In contrast, the α2 Na,K-ATPase is distributed in two distinct membrane pools. In the endplate membrane region (junctional pool), a smaller pool of the α2 Na,K-ATPase is localized, where it functionally and molecularly interacts with nicotinic acetylcholine receptors [24]. Most of the α2 Na,K-ATPase is expressed in the membrane of transverse tubule (extrajunctional pool), where it prevents the significant elevation of K^+^ upon muscle activity [21].

The Na,K-ATPase α subunit contains a highly specific binding site for cardiotonic steroids [25,26,27]. Plant alkaloid ouabain is a cardiotonic steroid which is suggested to be synthesized in the adrenal cortex and hypothalamus [28]. Accordingly, endogenous ouabain was shown to circulate in subnanomolar concentrations, but it can be elevated under various physiological and pathophysiological conditions [26,28,29,30,31]. High concentrations of ouabain are very toxic because it blocks the activity of Na,K-ATPase. However, because endogenous ouabain is usually detected at low concentrations, it is now suggested as a hormone [28]. Increasing evidence indicates a broad therapeutic potential of low ouabain concentrations in an inflammatory response, blood pressure regulation, and neural signaling [23,26,28,32,33]. In skeletal muscle, the chronic elevation of circulating ouabain has been shown to prevent some disuse-induced changes, including reductions in the Na,K-ATPase ion transport and membrane depolarization. An activation of inflammatory cytokine, IL-6 signaling, has been suggested to be involved in these preventive effects of ouabain [34,35].

In this study, we address the specific role of Na,K-ATPase in the IR-induced electrogenic changes in skeletal muscle, and test the preventive potential of exogenous ouabain against harmful effects of IR.

## 2. Results

### 2.1. Physiological Parameters of Rats

**Figure 1:**
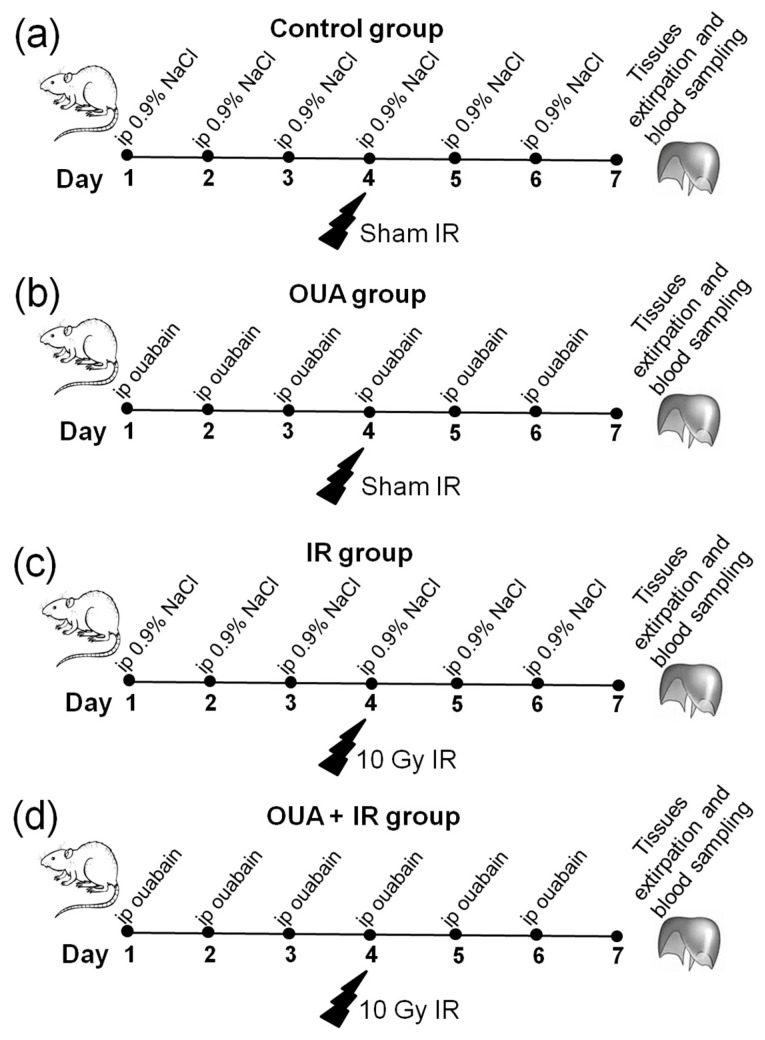
The study outline. (**a**–**d**) Rats were randomly divided into two groups and subjected to daily intraperitoneal (i.p.) injections of either 1 mL sterile 0.9% NaCl (vehicle)—(**a**,**c**), or ouabain (1 µg/kg body weight) dissolved in 1 mL sterile 0.9% NaCl (**b**,**d**). On the fourth day of injections, rats in these two groups were randomly divided into rats exposed to irradiation procedure (**c**,**d**) and sham-treated rats (**a**,**b**). Thus, there were four experimental groups: rats subjected to i.p. injections of saline without exposure to irradiation—‘Control’ group (**a**); rats treated with ouabain without exposure to irradiation—‘OUA’ group (**b**); vehicle-injected rats exposed to total-body X-ray irradiation (10 Gy)—‘IR’ group (**c**); and rats treated with ouabain and exposed to irradiation—‘OUA + IR’ group (**d**). The diaphragm muscles and colon were dissected, and blood was collected 72 h after exposure to irradiation.

**Figure 2:**
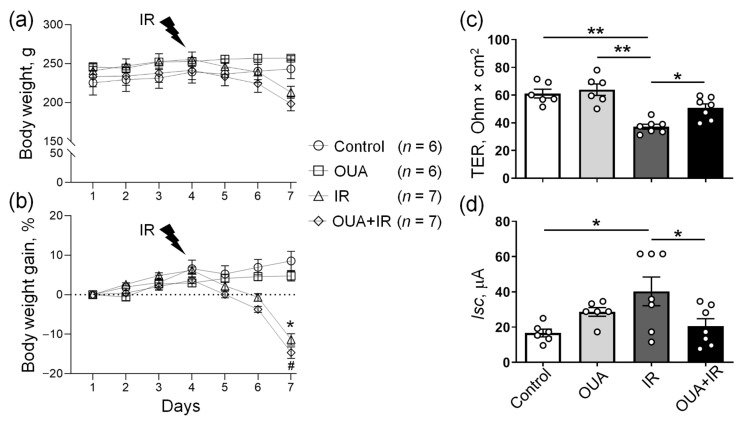
The effects of the chronic administration of exogenous ouabain (OUA), ionizing radiation (IR), and their combination (OUA + IR) on the physiological parameters of rats. (**a**,**b**) Daily body weight changes during the study in four experimental groups. Rats exposed to IR lost weight, whereas rats that were not exposed to IR gained weight over time. (**c**,**d**) Changes in the barrier and transport properties of rat colon epithelium were measured in the Ussing chamber. (**c**) Transepithelial electrical resistance (TER). (**d**) Short-circuit current (*Isc*). Measurements were performed after 5 min incubation in the Ussing chamber. For each rat, 3–4 fragments of the colon were examined. The number of rats corresponds to the number of symbols; *n* = 6−7. (**a**,**b**) * and #, *p* < 0.05 IR group vs. control group, and OUA + IR group vs. control group, respectively. (**c**,**d**) * and **, *p* < 0.05 and 0.01. Two-way ANOVA followed by Bonferroni multiple comparisons test.

The study design is presented in Figure 1a–d. Control rats gained weight during a study period, and chronic treatment with ouabain (1 µg/kg) did not affect weight changes in comparison with these controls (Figure 1b,d). Rats exposed to IR responded with anorexia, diarrhea, and exhibited body weight loss by 12% compared with the control rats, as previously reported [5]. Chronic ouabain treatment did not prevent IR-induced weight loss (Figure 2a,b).

The functional state of the colon tissue isolated from the same rats was evaluated as an additional physiological indicator to the body weight. Previously, it has been shown that IR dramatically affects the transport and barrier functions of the intestine, which plays an important role in maintaining body homeostasis [36]. The functional state of the colon was assessed in the Ussing chamber. As expected, IR significantly decreased transepithelial electrical resistance (TER) and increased short circuit current (*Isc*) values of the colon epithelium (Figure 2c,d). This suggests a violation of the barrier properties of epithelium (TER), as well as disturbance of passive and active transport across the epithelium (*Isc*) after IR exposure. Although chronic administration of ouabain alone had no significant effect on these parameters, after ouabain administration, IR-induced epithelium disturbances were not seen (Figure 2c,d). Thus, our findings suggest the ability of chronic ouabain administration to modulate the transport and barrier functions of the rat colon epithelium damaged by IR exposure.

### 2.2. IR Elevates the Serum Level of Ouabain, Activates Inflammation and Antioxidant Responses

**Figure 3:**
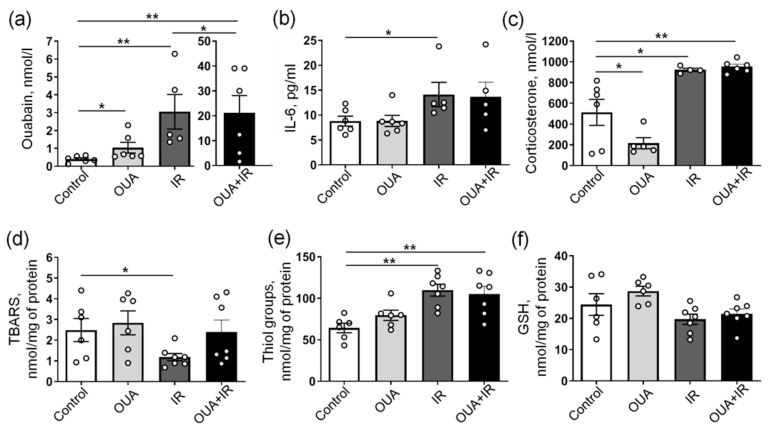
Biochemical responses to chronic administration of exogenous ouabain (OUA), ionizing radiation (IR), and their combination (OUA + IR). (**a**–**c**) Concentrations of ouabain, IL-6, and corticosterone measured in the blood serum, as indicated. (**d**) Thiobarbituric acid reactive substance (TBARS) concentrations, (**e**) the concentration of thiol groups, and (**f**) total glutathione (GSH) measured in homogenates of the diaphragm muscle. The number of rats corresponds to the number of symbols. * and **, *p* < 0.05 and 0.01 (two-way ANOVA followed by Bonferroni multiple comparisons test).

The concentration of endogenous ouabain in the blood serum of control rats was 0.40 ± 0.07 nM (*n* = 6). Similarly to previous studies [34,37], chronic administration of exogenous ouabain (1 μg/kg) significantly increased the concentration of serum ouabain to 1.04 ± 0.30 nM (*n* = 6) (Figure 3a). IR also increased the endogenous ouabain concentration to 3.1 ± 1.0 nM (*n* = 5). In rats chronically treated with exogenous ouabain and exposed to IR, the concentration of ouabain in blood serum was further dramatically elevated to 21.2 ± 6.9 nM (*n* = 6) (Figure 3a). This suggests that IR itself stimulates an increase in endogenous ouabain and that exogenous ouabain treatment followed by IR further amplifies this elevation.

In accordance with previous reports [9,10], IR significantly increased the serum level of IL-6 (Figure 3b), suggesting an inflammatory response. This significant elevation of IL-6 was not seen when IR was applied to rats chronically treated with exogenous ouabain. Importantly, the chronic administration of exogenous ouabain alone did not affect serum levels of IL-6 (Figure 3b). Elevated IL-6 is usually associated with the activation of the hypothalamic–pituitary–adrenal (HPA) axis [38]; therefore, we measured the serum concentration of corticosterone. Accordingly, IR also significantly increased the concentration of serum corticosterone (Figure 3c), suggesting an activation of the HPA axis and increased stress response [39]. Chronic ouabain administration significantly reduced serum corticosterone under control conditions, but it failed to prevent IR-induced elevation in serum corticosterone level (Figure 3c).

Both inflammation and HPA axis activation are known to be associated with increased oxidative stress [40]. The redox regulation of the Na,K-ATPase is also well documented [41]. Both IR-induced elevations and reduction in lipid peroxidation were previously demonstrated, and these changes were tissue-, dose-, and time-dependent [42]. Therefore, we assessed the lipid peroxidation level using a thiobarbituric acid reactive substances (TBARSs) assay in the diaphragm muscle. We found that IR significantly reduced the level of TBARS (Figure 3d), suggesting the reduced level of lipid peroxidation. The TBARS level was unaffected by chronic ouabain treatment. However, the IR-induced reduction in TBARSs was not seen after ouabain administration and with a combination of IR and ouabain, the TBARS level was not significantly different from both the control group and the IR group (Figure 3d).

This surprising IR-induced reduction in TBARS can be a result of antioxidant system activation. Accordingly, we found elevated levels of thiol groups upon IR exposure; however, this change in thiol groups was not affected by chronic ouabain treatment (Figure 3e). No changes in total glutathione were seen at any intervention (Figure 3f). Altogether, these results suggest that IR induced inflammation and lipid peroxidation, but this was associated with potentiated antioxidant response. An increased concentration of serum ouabain might contribute to this association.

### 2.3. IR Decreases Electrogenic Contribution of the α2 Na,K-ATPase and Depolarizes Sarcolemma

**Figure 4:**
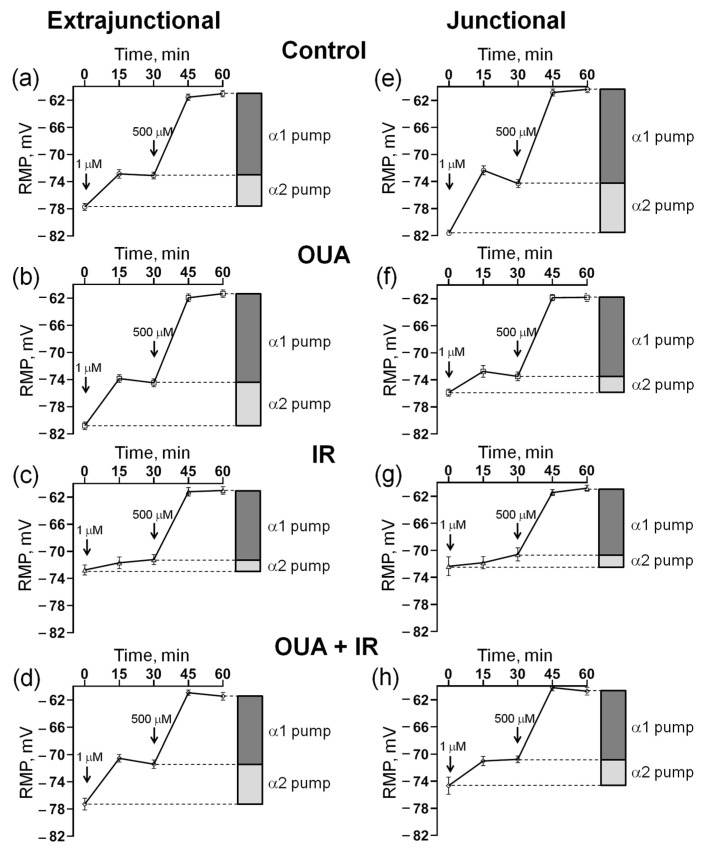
Measurement of the resting membrane potential (RMP) and estimation of the electrogenic contribution of α2 and α1 Na,K-ATPase isozymes with the sequential addition of 1 µM and 500 µM ouabain to the bath solution (indicated by arrows). Measurements were performed in the extrajunctional (**a**–**d**) and junctional (**e**–**h**) regions of the sarcolemma of diaphragm muscles. Rats were subjected to 6 days of injections of either 1 mL sterile 0.9% NaCl (vehicle) or ouabain (1 µg/kg body weight) dissolved in 1 mL sterile 0.9% NaCl. (**a**,**e**) Control group, where rats were subjected to injections of vehicle (0.9% NaCl). (**b**,**f**) OUA group, where rats were subjected to injections of ouabain. (**c**,**g**) IR group, where rats were subjected to vehicle (0.9% NaCl) and exposed to a single total-body X-ray irradiation (10 Gy). (**d**,**h**) OUA + IR group, where rats were chronically treated with ouabain and exposed to IR. Each data point corresponds to the averaged RMP measured in at least 100 muscle fibers of 6−7 diaphragm muscles.

For each muscle, the initial RMP value was first recorded; then, increasing concentrations of 1 μM and 500 μM were sequentially added to the bath solution to estimate the electrogenic contribution of α2 and α1 Na,K-ATPase, respectively (see the Methods). These procedures were performed on both the extrajunctional (Figure 4a–d) and junctional (Figure 4e–h) regions of the sarcolemma. Importantly, with the complete inhibition of the Na,K-ATPase activity by 500 μM ouabain, the same RMP (~−61 mV) was established in all experimental groups (Figure 4a–h).

**Figure 5:**
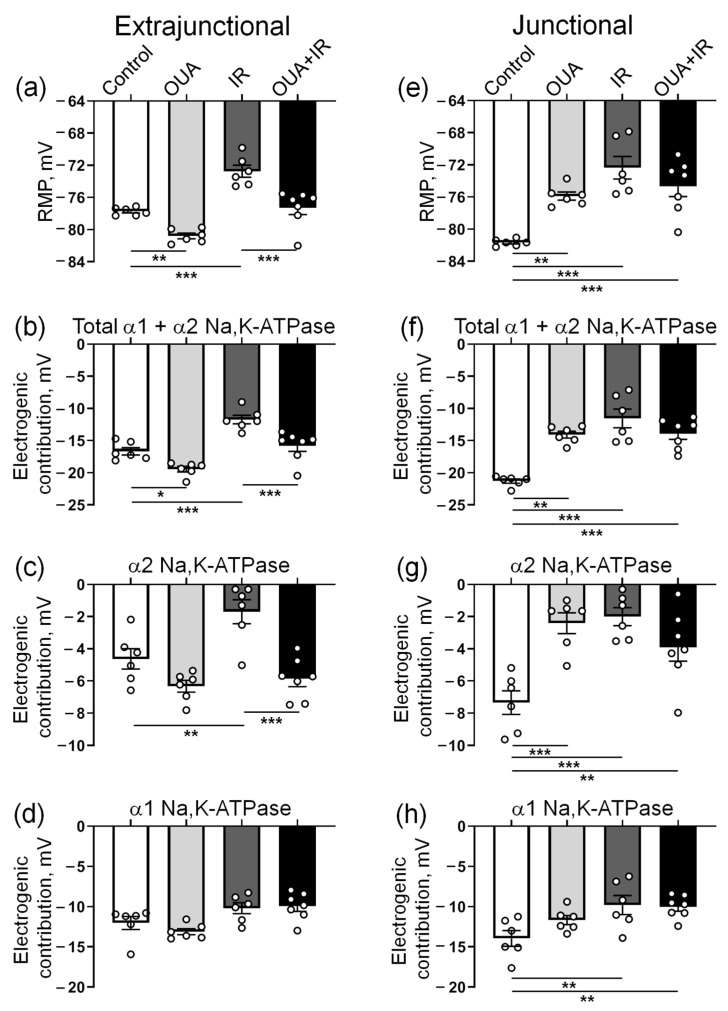
The Na,K-ATPase-dependent membrane potential changes in the extrajunctional (**a**–**d**) and junctional (**e**–**h**) regions of the sarcolemma of diaphragm muscle from control rats, rats chronically treated with ouabain (OUA), rats exposed to ionizing radiation (IR), and rats chronically treated with ouabain and exposed to IR (OUA+IR). (**a**,**e**) Resting membrane potential (RMP). (**b**,**f**) Total electrogenic contribution of the Na,K-ATPase was measured as the sum of electrogenic activities of the α2 and α1 isozymes of Na,K-ATPase. (**c**,**g**) Electrogenic contribution of the α2 Na,K-ATPase as membrane potential changes in the presence of 1 µM ouabain. (**d**,**h**) Electrogenic contribution of the α1 Na,K-ATPase as membrane potential changes in response to the sequential addition of 500 µM ouabain to the bath solution. The number of muscles corresponds to the number of symbols; *n* = 6−7 in all groups. *, **, and ***, *p* < 0.05, 0.01 and 0.001 (two-way ANOVA followed by Bonferroni multiple comparisons test).

We found that 72 h after IR intervention, the RMP in the extrajunctional membrane region of the diaphragm was depolarized from −77.7 ± 0.2 mV (*n* = 6) in control muscles to −72.7 ± 0.7 mV (*n* = 6) in the muscles from rats exposed to IR (Figure 5a). Accordingly, the total contribution of the Na,K-ATPase to the RMP was decreased from −16.7 ± 0.6 mV to −11.7 ± 0.7 mV (Figure 5b). The electrogenic contribution of α2 Na,K-ATPase was decreased from −4.6 ± 0.6 mV up to −1.7 ± 0.7 mV (Figure 5c), whereas contributions of the α1 Na,K-ATPase were not significantly changed (Figure 5d). IR also depolarized the RMP in the junctional sarcolemma region, which was associated with a predominant decrease in the electrogenic contribution of the α2 Na,K-ATPase (Figure 5e–h).

**Figure 6:**
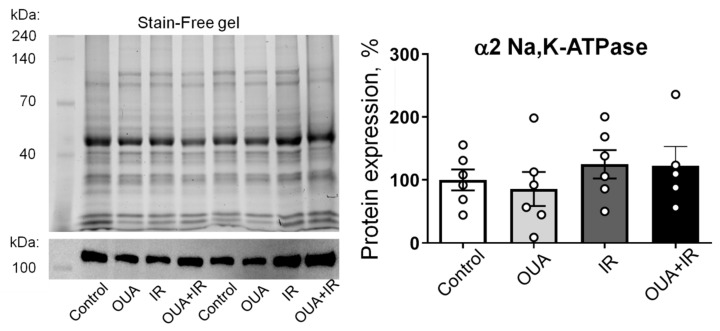
Ionizing radiation and 6-day ouabain treatment did not induce any significant changes in the α2 Na,K-ATPase abundance in the rat diaphragm muscle. The protein content of the α2 Na,K-ATPase in muscle from control rats, rats chronically treated with ouabain (OUA), rats exposed to ionizing radiation (IR), and rats chronically treated with ouabain and exposed to IR (OUA+IR). Left panel shows representative total protein load detected with Stain-Free gel and the corresponding Western blots for the α2 Na,K-ATPase, used for semi-quantification of α2 isoform abundance, i.e., a ratio between a specific α2 Na,K-ATPase band intensity over total protein load for the same probe. The right panel is the averaged results of semi-quantification of the α2 isoform protein abundance. The number of muscles corresponds to the number of symbols; *n* = 5−6 in all groups.

We tested the α2 Na,K-ATPase total protein contents measured in homogenates of whole diaphragm muscles; no significant changes were found in all studied groups compared with the control group (Figure 6).

This is in contrast with our functional results on the electrogenic contribution of the α2 Na,K-ATPase. However, our Western blot experiments detected the total cellular content of the α2 Na,K-ATPase, whereas the electrogenic contribution is only provided by the membrane pool of the Na,K-ATPase. This suggests that membrane abundance and activity, but not the expression of α2 Na,K-ATPase, may be modified by the interventions in this study.

### 2.4. IR-Induced Loss in the Electrogenic Contribution of the α2 Na,K-ATPase and Sarcolemma Depolarization Was Not Seen after Chronic Ouabain

Similarly to previous observations [34], chronic administration of ouabain (1 μg/kg) significantly hyperpolarized the RMP in the extrajunctional membrane region (Figure 5a). Moreover, the IR-induced RMP depolarization was not seen after chronic ouabain administration. This effect of ouabain was accompanied by increases in the total electrogenic contribution of Na,K-ATPase (Figure 5b), where the changes were predominantly attributed to the α2 isozyme (Figure 5c), while the contribution of α1 Na,K-ATPase was unchanged (Figure 5d). Notably, these changes were not associated with the changes in the α2 Na,K-ATPase protein content (Figure 6).

In the junctional sarcolemma regions, chronic ouabain administration depolarized sarcolemma, as reported previously [34]. However, in contrast to the extrajunctional membrane region, chronic ouabain treatment did not affect the IR-induced reduction in electrogenic contribution of the α2 Na,K-ATPase, and thus, IR-induced membrane depolarization (Figure 5e–h). These observations are in agreement with our previous report showing that the extrajunctional and junctional pools of the α2 Na,K-ATPase are differentially regulated [22,34].

## 3. Discussion

In this study, we found that a single IR exposure has isoform-specific detrimental action on the Na,K-ATPase function in skeletal muscles, and that this effect can be rescued by chronic exogenous ouabain administration. The observed reduction in electrogenic contribution of the α2 Na,K-ATPase isozyme was not associated with changed protein abundance, but rather represents either its changes in subcellular localization or inhibitory influence on the enzyme activity of free radicals and possibly associates with inflammation. An ability of chronic exogenous ouabain to elevate the concentration of endogenous ouabain in the blood serum and the lack of IR-induced reduction in lipid peroxidation in the presence of elevated serum ouabain may be a positive effect of ouabain in skeletal muscle at IR exposure.

Exposing cells to IR is known to cause immediate free radical formation leading to ROS generation, and thus, cell damage [10]. ROS are also present in healthy cells serving multiple signaling functions, but the body’s antioxidant systems, including thiols, control their activity by neutralizing the excessive free radicals. IR-induced increases in free radicals were previously shown to potentiate lipid peroxidation, which leads to cell membrane damage [43]. Changes in membrane lipid composition could have dramatic consequences for Na,K-ATPase enzymatic activity [44]. Accordingly, an increase in the concentration of lipid peroxidation products (TBARSs) was previously correlated with inhibition of the Na,K-ATPase [45,46,47]. It might, therefore, be surprising that TBARSs were found reduced in this study upon the IR exposure. However, this was associated with increased reduced thiol groups, which contribute to antioxidant responses. Notably, both IR-induced elevations and reductions in TBARS production were previously found, and these changes were tissue-, dose-, and time-dependent [42]. IR exposure and free radicals induce apoptosis and cell necrosis, which, in turn, activate inflammation and inflammatory cytokine release [9,10,48]. In the absence of mechanisms suppressing these responses, chronic inflammation may continue for a long time, as it is evident in this study where IL-6 was elevated 72 h after the IR. Altogether, our results suggest that IR activated the redox system, possible because of the initial elevation of free radicals, and this redox system was probably still overactive 72 h after IR exposure.

Previous reports on the IR-induced structural disintegration of the lipid bilayer [49,50] suggest that changes in membrane potential can be independent of the electrogenic Na,K-ATPase contribution. However, the same RMP (~−61 mV) was established in both control and IR-treated muscles, when Na,K-ATPase activity was completely inhibited with 500 μM ouabain. This confirms that the IR-induced alterations in the RMP are not a simple result of unspecific membrane disintegration. Our results clearly suggest that IR causes membrane depolarization due to a decrease in the electrogenic contribution of the α2 Na,K-ATPase. Similar arguments can be provided for a specific effect of the chronic administration of ouabain on the RMP and α2 Na,K-ATPase electrogenic contribution.

The negative effects of IR on the Na,K-ATPase activity have been reported in various tissues, including rabbit lens [12], rabbit kidney [13], and rat kidney [5]. The effect of IR on the Na,K-ATPase function in skeletal muscle has not yet been studied. The suppression of Na,K-ATPase by IR was previously suggested to be mediated via IR-induced free radicals [13]. We also suggested that this is the case in our study. In kidney, a single exposure to IR (25 Gy) caused alternations in the oxidative status and decreased the activity of Na,K-ATPase [5]. Importantly, kidney epithelial cells express only the α1 Na,K-ATPase, and its reduced activity was suggested to be due to decreased membrane abundance of the Na,K-ATPase as a result of diminished incorporation into the plasma membrane. Moreover, IR also significantly decreased the activity and abundance of the Na,K-ATPase in rat cardiac muscle; however, the isoform specificity of these changes was not addressed here [51].

In the current study, chronic ouabain administration, IR (10 Gy), or both interventions changed the α2 Na,K-ATPase electrogenic contribution without changes in its protein abundance. This suggests that these interventions can modulate the trafficking between α2 Na,K-ATPase intracellular pool and the sarcolemma and/or directly modulate the enzyme activity. This possibility is supported by previous findings that chronic ouabain treatment increased the number of [^3^H] ouabain binding sites, RMP, and the Na,K-ATPase electrogenic contribution in cultured rat myotubes, suggesting an increase in the Na,K-ATPase membrane abundance [52].

Although we cannot exclude the minor effect of IR on skeletal muscle α1 Na,K-ATPase in our study, the lack of changes in electrogenic contribution of the α1 Na,K-ATPase after IR exposure does not support it. In fact, reduced stable integration of the α2 Na,K-ATPase into the lipid bilayer compared with the α1 and α3 isoforms was reported [53,54], which may be the reason for isoform specificity of the IR effect in skeletal muscle.

The IR-induced loss of the catalytic activity of α2 Na,K-ATPase also cannot be excluded. The total-body IR caused a significant decline in the activity of renal Na,K-ATPase, despite the protection of kidneys from irradiation by lead plates [5]. This may be a result of deteriorated global homeostasis, e.g., increased oxidative stress. It is possible that some factors which changed distantly from the diaphragm were delivered with the blood and affected the muscle. In fact, it has been shown that IR at a dose of 10 Gy is sufficient to induce oxidative stress in the mouse diaphragm, accompanied by muscle fatigue [3]. Lipid peroxidation can affect plasma membrane lipids and disorganize the arrangement of lipid bilayer, leading to changes in membrane integrity and fluidity [49,50]. This can cause structural disintegration of the plasma membrane and reduction in the function and activity of integral proteins, including the Na,K-ATPase [47]. Moreover, Na,K-ATPase is a cellular protein which is highly sensitive to free radicals. In fact, the enzymatic activity of Na,K-ATPase was shown to be impaired in the presence of free radicals [55].

Previous studies reported that the administration of ouabain in rats modulates membrane lipid composition [56]. Moreover, a specific interactions between the α2 Na,K-ATPase and membrane cholesterol were assumed: the loss of membrane cholesterol reduced the transport activity of α2 Na,K-ATPase, and vice versa [57]. The IR-associated increase in cholesterol levels was recently shown to be due to modulation of the cholesterol biosynthetic pathway [58]. These facts open a possibility that ouabain minimized the deleterious effects of IR on the α2 Na,K-ATPase activity via the modulation of lipid composition, which otherwise is damaged by IR.

Although ouabain is known to specifically inhibit the Na,K-ATPase, it has also been shown to activate both α1 [37,59,60] and α2 [61] isoforms at concentrations comparable to the endogenous ouabain level. Endogenous ouabain has been characterized as a hormone synthesized in the zona glomerulosa cells of the adrenal cortex from cholesterol [28]. Accordingly, we also detected it in rat serum samples. Little is known about the regulation of endogenous ouabain synthesis and release, although it has been suggested to be regulated by the sympathetic nervous system and stress–hormone axis [28], which are also known to be activated by IR [38]. In accordance with previous studies [34,37], we have found that the administration of exogenous ouabain elevated the circulating ouabain level by approximately twofold. Although this could be a result of the chronic administration of ouabain that was not yet cleared, the feedback mechanism cannot be excluded. It has previously been shown that an elevation of ouabain can activate the sympathetic nervous system [62] which, in term, can stimulate the release of ouabain from adrenal glands [28,63].

We have also detected the elevation of endogenous ouabain upon IR treatment, and although the mechanism behind it is unclear, the involvement of stress response, as validated by increased serum corticosterone levels in this study, cannot be excluded. Accordingly, a combination of chronic exogenous ouabain treatments and IR further elevated the concentration of circulating ouabain up to ~20 nM. Importantly, the ability of ouabain at 10−20 nM concentrations to hyperpolarize extrajunctional skeletal muscle membrane was previously shown [34].

Notably, in contrast to the suppressive action of free radicals on the Na,K-ATPase, free radicals have been suggested to be an important component of the oxidative stress amplification loop [64]. Thus, binding of ouabain to the Na,K-ATPase has been suggested to activate pathways that can increase the generation of mitochondrial ROS, which, in turn, can further modify the Na,K-ATPase and potentiate this signaling [65]. The Na,K-ATPase-dependent ROS pathway is suggested to be potentiated upon direct carbonylation by ROS [66]. In the current study, the observed normal level of lipid oxidation in the IR exposed rats, which were pre-treated with ouabain, may be due to activation of this Na,K-ATPase/ROS amplification loop by ouabain [64] and IR-induced reduction in lipid peroxidation because of potentiated antioxidant responses.

We should note that cardiotonic steroid ouabain can affect cardiac function and blood pressure [67]. We did not assess these parameters here, but suggest that these are of minor significance. It has previously been shown that blood pressure did not change in rats treated with concentrations of ouabain similar to those this study for 4 days. Only after 8 days of chronic treatment, a significant increase in blood pressure was reported; however, without any change in heart rate [37]. Thus, we suggest that, in our study, during the first 4 days of ouabain treatment (prior IR exposure), blood pressure was not significantly changed. Ex vivo analyses were performed 6 days after ouabain administration; therefore, we do expect the changes in cardiovascular parameters of significance, although we cannot exclude this. Several studies have suggested that relatively low doses of IR can affect the cardiovascular system [68]; however, this was not addressed in this study, nor were the combined effects of ouabain and IR. This calls for future experiments. Moreover, the differential effect of IR on both α1 and α2 Na,K-ATPase remains to be studied. Although our functional analysis indicates the primary role of α2 Na,K-ATPase, the effects of both ouabain administration [37] and IR [5] on renal α1 Na,K-ATPase were previously suggested.

Could ion transport other than Na,K-ATPase be involved in the effects of IR and ouabain administration? It has been reported that ouabain can increase the late sodium current and reverse the Na,Ca exchange current in ventricular myocytes, with significant effects at concentrations of about 1 μM and more [69,70,71]. Thus, it seems unlikely that long-term treatment with ouabain in our study could affect voltage-gated sodium channels that are functionally present in skeletal muscle, because relatively high concentrations of ouabain (compared with circulating ouabain measured in this study) are required to mediate these effects.

However, it should be noted that the production of free radicals, which is characteristic of IR [4], increases the magnitude of late sodium current, accompanied by intracellular sodium and calcium overload [72,73]. Moreover, previous studies have shown that endogenous ouabain levels correlate positively with blood pressure, and sodium channels mediate an elevation of ouabain concentration and blood pressure in rats [74]. It is therefore possible that the IR-induced generation of free radicals and the increased activity of sodium channels may provoke a subsequent elevation of endogenous ouabain release, as previously suggested for ouabain homeostasis in the brain [74].

Previous studies on the electrogenic contribution of Na,K-ATPase in the rat skeletal muscle cells used high concentrations of ouabain and K^+^-free solutions, which both inhibit all subunits of the Na,K-ATPase, as well as cooling, which alters enzyme activity. All these acute interventions decrease membrane potential from approximately −80 mV level to values of approximately −60 to −65 mV [24,75,76]. This membrane potential in a complete absence of the Na,K-ATPase electrogenic transport is solely determined by membrane permeability and ionic gradients in accordance with the Goldman–Hodgkin–Katz equation [77,78]. These observations indicate that the total electrogenic contribution of Na,K-ATPase in these preparations is about −15 to −20 mV [24,75,76].

In our study, in all experimental conditions (control, chronic ouabain administration, IR, or both interventions), the acute administration of 500 μM ouabain, which completely inhibits the Na,K-ATPase activity, also depolarized RMP to about −61 mV. This suggests that observed membrane potential changes are likely due to changes in the electrogenic contribution of Na,K-ATPase rather than a result of changes in other ion transport. However, we measured the electrogenic contribution of the α1 and α2 isoforms of Na,K-ATPase in isolated resting non-contracting muscles in vitro. In contrast, under physiological conditions, skeletal muscles generate action potentials, and IR can alter the properties of sodium channels and action potentials, similar to how it affects brain tissue (for review [79]), and thus, influences the background sodium conductance. We believe that further studies are needed to examine this possibility.

**Figure 7:**
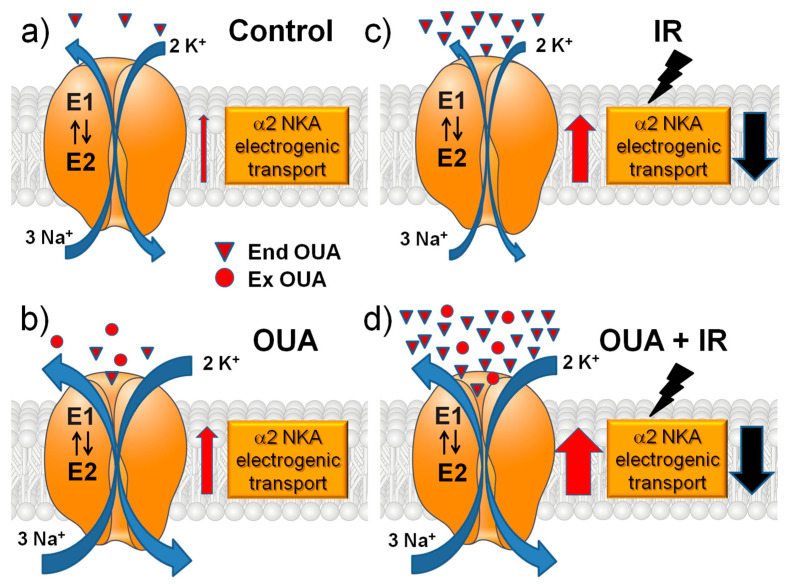
A schematic presentation of the main interventions in this study and their modulatory effects on the α2 Na,K-ATPase electrogenic transport. The role of changes in sodium membrane conductance is discussed (see the text). The effects of chronic ouabain (OUA) administration, ionizing radiation (IR), or their combination (OUA + IR) in the rat diaphragm muscle were studied. (**a**) Control group: relatively low concentration of endogenous ouabain (triangles, End OUA). (**b**) Chronic administration of exogenous ouabain (circles, Ex OUA) doubles the concentration of ouabain in circulation. (**c**) IR itself greatly increases the concentration of endogenous ouabain. (**d**) Dramatic elevation of ouabain concentration under chronic administration of exogenous ouabain followed by IR intervention. Red and black arrows show the increase and decrease in the α2 Na,K-ATPase electrogenic transport, respectively.

Overall, we can schematically summarize our results in Figure 7.

## 4. Materials and Methods

### 4.1. Animals and Irradiation Procedure

Experiments were performed on age- and weight-matched male Wistar rats (10 weeks, ~230 g starting weight) housed in a temperature- and humidity-controlled animal facility with food and water ad libitum. All animal experiments were performed in accordance with the Guide for the Care and Use of Laboratory Animals [80]. The experimental protocol was in accordance with the requirements of the EU Directive 2010/63/EU for animal experiments and approved by the Bioethics Committee of St. Petersburg State University no. 131-03-5 (issued 13-12-2017).

The study design is presented in Figure 1a–d. Briefly, rats were subjected to 6 days of intraperitoneal injections of either 1 mL sterile 0.9% NaCl (vehicle)—‘Control’ group, or ouabain (1 µg/kg body weight) dissolved in 1 mL sterile 0.9% NaCl (‘OUA’ group). On the fourth day of injections, rats from ouabain- and vehicle-treated groups were randomly subdivided into the groups subjected to the IR procedure or assigned as sham groups, i.e., control (*n* = 6) and OUA (*n* = 6) groups (Figure 1a,b). Rats (*n* = 7) treated with vehicle and then exposed to total-body X-ray irradiation were assigned to the ‘IR’ group (Figure 1c). Rats (*n* = 7) treated with ouabain and then exposed to IR were assigned to the ‘OUA + IR’ group (Figure 1d).

Rats were exposed to one-time total-body X-ray irradiation (10 Gy) using the RUM-17 orthovoltage therapeutic X-ray unit (MosRentgen). During the procedure, freely respirating rats were restricted in a closed plexiglass box. The focal length of the X-ray tube was 50 cm, with a dose rate of 0.31 Gy/min. An absorbed dose was controlled using an individual dosimeter, and the results were interpretated with a GO-32 measuring device (Spetsoborona). For the sham irradiation procedure, rats were placed in the same restriction box under the switched-off X-ray tube for the same exposure time. Rat body weights were measured at the beginning of protocol and daily during the experiment.

Rats were euthanized 72 h after exposure to IR by the intraperitoneal injection of a tribromoethanol overdose (750 mg/kg) followed by decapitation. The diaphragm muscle and colon were isolated and mixed blood was collected. Freshly isolated diaphragm muscle and colon fragments were immediately used for electrophysiological measurements. Some muscles were also snap-frozen in liquid nitrogen and then stored at −80 °C for later biochemical and expression assays.

### 4.2. Biochemical Analyses of Blood and Tissue Samples

Serum concentrations of ouabain were measured with the ELISA Kit for Ouabain (CEV857Ge, Cloud-Clone corp., Katy, TX, USA). Serum IL-6 levels were estimated using the High Sensitive ELISA kit with detection range 1.56–100 pg/mL (HEA079Ra, Cloud-Clone Corp., Katy, TX, USA). Serum corticosterone levels were estimated using the ELISA kit (EIA-4164, DRG International Inc., USA). The assay’s procedures were conducted in accordance with the manufacturer’s protocols, and the light absorbance was measured at 450 nm with a spectrophotometric microplate reader SPECTROstar Nano (BMG Labtech, Ortenberg, Germany). The concentrations of ouabain, IL-6, and corticosterone were quantified based on the respective standard curves.

Lipid peroxidation products in the muscle tissue lysate were estimated using the thiobarbituric acid reactive substances (TBARS) assay, as described previously [81]. The optical density of TBARSs was measured using a spectrophotometric microplate reader SPECTROstar Nano (BMG Labtech, Germany) at 535 nm with a reference optical density wavelength of 600 nm at room temperature. TBARS concentrations were expressed in nmol per mg of total protein in the sample.

The amount of reduced thiol groups in cytosolic proteins was analyzed as a marker of redox status by adding 150 μL of 5,5’-dithiobis-(2-nitrobenzoic acid) derivatization solution (52.5 µg/mL) to 50 µL of the cytosolic fraction in 0.1 M potassium phosphate/1mM ethylenediaminetetraacetic acid buffer (pH 7.4) [82]. A spectrophotometric microplate reader SPECTROstar Nano (BMG Labtech, Ortenberg, Germany) was used to measure the optical density at 412 nm. The concentration of thiol groups was quantified using a reduced glutathione standard curve, and expressed as nmol of cysteine per mg of total protein.

The naphthalene-2,3-dicarboxaldehyde method was performed for measurements of total glutathione [83]. To deproteinize 80 μL of cytosolic fractions, it was diluted with an equal volume of 2 M perchloric acid, incubated for 5 min, and centrifuged at 3000× *g* for 5 min. Then, 100 μL of the supernatants was neutralized with 80 μL of 2 M potassium hydroxide, incubated for 5 min, and centrifuged again at 3000× *g* for 5 min. Total glutathione concentrations were detected using 70 μL supernatants mixed with 10 μL of reduction agent tris(2-carboxyethyl)phosphine hydrochloride (40 mM) and incubated for 10 min at room temperature [84]. Then, 20 μL samples were transferred in duplicates to a 96-well plate and incubated in the darkness at room temperature for 25 min. Fluorescence intensity was measured (472 nm excitation/528 nm emission) using a Typhoon FLA 9500 high-resolution laser scanner (GE, Boston, MA, USA). Total glutathione concentrations were calculated from the reduced glutathione standard curve. Total glutathione concentrations were expressed as nmol per mg of total protein.

Total protein in the samples was measured using the Pierce Rapid Gold Bicinchoninic Acid Protein Assay Kit (Thermo Fisher Scientific, Waltham, MA, USA), according to the manufacturer’s protocol.

### 4.3. Membrane Potential Recording

The freshly isolated whole left hemi-diaphragm (~15 mm width) containing tendon, costal parts, and the nerve stump was dissected [85]. Tendon and costal parts were pinned in a Sylgard plate that was continuously perfused with physiological solution (in mM: NaCl, 137; KCl, 5; CaCl_2_, 2; MgCl_2_, 2; NaHCO_3_, 24; NaH_2_PO_4_, 1; glucose, 11; pH 7.4). The solution was continuously bubbled with 95% O_2_ and 5% CO_2_ and maintained at 28 °C. The RMP was recorded from the surface fibers using intracellular glass microelectrodes. The RMP recordings were made in the extrajunctional membrane regions within ~2 mm from visually identified terminal branches of the phrenic nerve, or directly near the nerve terminals (junctional membrane regions), as described previously [86]. In each muscle, RMPs were recorded from ~25 to 35 different muscle fibers for each (junctional and extrajunctional) membrane region during a 5–10 min period. For each muscle, the RMPs were averaged, and this value was used for statistical analysis as a single experimental value and for final mean calculation in the experimental series.

### 4.4. Electrogenic Contribution of Different Isozymes of the Na,K-ATPase

Alternating-gate models of the Post–Albers transport cycle of the Na,K-ATPase pump suggest that during each active cycle, 3 Na^+^ ions are pumped out of the cell in exchange for 2 incoming K^+^ ions for every ATP molecule. Due to this non-equilibrium exchange, RMP is further shifted towards a more negative value, called the “electrogenic contribution” of Na,K-ATPase [75]. The Na,K-ATPase electrogenic contribution is determined in isolated rodent muscles by measuring RMP changes in response to the inhibition of different isoforms of the Na,K-ATPase with different ouabain concentrations. This sensitive real-time method to estimate the electrogenic contribution of Na,K-ATPase transport to membrane potential of skeletal muscle was characterized previously [24,76]. Due to the more than 100-fold difference in ouabain affinities of rodent α1 and α2 Na,K-ATPase isoforms [25], 1 μM ouabain inhibits only α2 isoform, and the elevation of ouabain concentration to 500 μM completely inhibits both α1 and α2 isoforms [24,76]. The electrogenic contribution of the α2 isozyme was estimated as the change in mean RMP after 30 min incubation with 1 µM ouabain. The contribution of α1 isozyme was the RMP difference in the presence of 1 µM ouabain and after 30 min incubation with 500 µM ouabain.

### 4.5. Western Blot Assays

After the homogenization of skeletal muscles in a lysis buffer (in mM: Tris, 20; NaCl, 150; Triton X-100, 1; EDTA, 5; tween-20, 0.1; protease inhibitors cOmplete mini tablets (Roche, Mannheim, Germany, pH 7.6), samples were incubated on ice for 30 min, then sonicated and centrifugated for 15 min at 13,000× *g* at 4 °C (Eppendorf, Hamburg, Germany). Total protein contents in the supernatants were measured with a Pierce Rapid Gold Bicinchoninic Acid Protein Assay Kit (Thermo Fisher Scientific, Waltham, MA, USA), according to the manufacturer’s protocol, using a spectrophotometric microplate reader SPECTROstar Nano (BMG Labtech, Ortenberg, Germany). Equal amounts of total protein were heated for 10 min at 37 °C with a 4x Laemmli buffer.

The total proteins were loaded on 10% Stain-Free gels and separated by SDS-PAGE (sodium dodecyl sulfate–polyacrylamide gel electrophoresis). Then, proteins were transferred to the PVDF membrane (Bio-Rad, Hercules, CA, USA). After blocking for 2 h with 5% skimmed milk at room temperature, the membranes were incubated overnight at 4 °C with 2.5% skimmed milk and monoclonal rabbit antibody against the α2 isoform (1:1000, MA5-37905, Thermo Fisher Scientific, Waltham, MA, USA). The next day, after intensive washing, the membranes were incubated for 45 min with 2.5% skimmed milk and horseradish-peroxidase-conjugated anti-rabbit secondary antibody (1:10,000, AB205718, Abcam, Eugene, OR, USA). After washing, bound antibody was detected with an enhanced chemiluminescence kit (Bio-Rad, Hercules, CA, USA). A luminescence imager Chemi-Doc XRS + Imaging System (Bio-Rad, Hercules, CA, USA) was used to measure band intensities that were normalized using Image Lab 6.1 Software (Bio-Rad, Hercules, CA, USA) to the total protein load in the same sample measured in the membrane prior to incubation with antibody.

### 4.6. Measurement of Electrophysiological Characteristics of the Colon

Samples of rat colon were obtained simultaneously with the diaphragm muscles from the same animals. Barrier properties of the epithelium (transepithelial electrical resistance, TER) and passive and active transport through the epithelium (short-circuit current, Isc) were estimated in the Ussing chamber, as described previously [36]. Briefly, colon fragments were mounted in Ussing chambers (exposed area: 0.13 cm^2^), and bathed with solution (in mM: NaCl, 119; KCl, 5; CaCl_2_, 1.2; MgCl_2_, 1.2; NaHCO_3_, 25; Na_2_HPO_4_, 1.6; NaH_2_PO_4_, 0.4; d-glucose, 10; pH 7.4). The solution was bubbled with 95% O_2_ and 5% CO_2_ and maintained at 37 °C. TER was calculated using Ohm’s law and voltage changes to 10 μA current. The registration of Isc was carried out when the voltage was fixed at zero level (0 mV). For each rat, 3–4 fragments of the colon were examined, and the data were averaged.

### 4.7. Statistics

Data are presented as the mean ± SEM. The difference between means was calculated using two-way ANOVA followed by Bonferroni multiple comparisons test. Statistical analysis was performed using GraphPad Prism 8 software (GraphPad; San Diego, CA, USA). A probability value of *p* < 0.05 was considered statistically significant. StatMate ver. 2 (GraphPad) was used to estimate the sample size to compare two means. Sample sizes of 5−6 were sufficient to detect a minimum difference in means within any measurement with a 5% error (i.e., *p* < 0.05) and a power of 0.8 (80% chance to correctly determine the difference between groups).

## 5. Conclusions

IR is used in many clinical settings, including many medical imaging modalities and cancer treatment. However, IR can also damage living cells, but the cells can also resist the deleterious effects of IR. IR can harm the cell in several ways, e.g., free radical generation and direct damage of DNA and other molecules; therefore, the body has multiple defense lines against it. This study suggests that elevations of circulating ouabain can minimize IR-induced skeletal muscle dysfunction. We suggest that protective effects of ouabain may be elevated by stress responses to IR as well as by exogenous administration of ouabain. The sufficiently high level of circulating ouabain is proposed to counterbalance some IR-induced changes in lipid peroxidation level and the α2 Na,K-ATPase activity in skeletal muscle membrane. Thus far, the question of the possible participation in the effects of IR and ouabain interventions of ion transport other than the Na,K-ATPase remains unresolved. It should also be noted that the densitometry method may not be sufficiently sensitive to assess relatively small changes in protein content. This limitation should be taken into account when considering the mechanism of changes in the functional contribution of the Na,K-ATPase. Altogether, although the detailed mechanism behind ouabain and IR action remains to be elucidated, our findings suggest its future therapeutic potential in the prevention of IR deleterious effects.

## Data Availability

The data that support the findings of this study are available from the corresponding author upon reasonable request.
